# Bayesian Estimation of Geometric Morphometric Landmarks for Simultaneous Localization of Multiple Anatomies in Cardiac CT Images

**DOI:** 10.3390/e23010064

**Published:** 2021-01-02

**Authors:** Byunghwan Jeon, Sunghee Jung, Hackjoon Shim, Hyuk-Jae Chang

**Affiliations:** 1 School of Computer Science, Kyungil University, Gyeongsan 38428, Korea; bhjeon@kiu.kr; 2 CONNECT-AI R&D Center, Yonsei University College of Medicine, Seoul 03722,Korea; sh.jung@yonsei.ac.kr (S.J.); hjshim@yuhs.ac (H.S.); 3 Division of Cardiology Department of Internal Medicine, Yonsei University College of Medicine, Seoul 03722, Korea

**Keywords:** Bayesian estimation, morphometric, pulmonary vein, left atrial appendage, geometric relation

## Abstract

We propose a robust method to simultaneously localize multiple objects in cardiac computed tomography angiography (CTA) images. The relative prior distributions of the multiple objects in the three-dimensional (3D) space can be obtained through integrating the geometric morphological relationship of each target object to some reference objects. In cardiac CTA images, the cross-sections of ascending and descending aorta can play the role of the reference objects. We employed the maximum a posteriori (MAP) estimator that utilizes anatomic prior knowledge to address this problem of localizing multiple objects. We propose a new feature for each pixel using the relative distances, which can define any objects that have unclear boundaries. Our experimental results targeting four pulmonary veins (PVs) and the left atrial appendage (LAA) in cardiac CTA images demonstrate the robustness of the proposed method. The method could also be extended to localize other multiple objects in different applications.

## 1. Introduction

As a type of arrhythmia, atrial fibrillation (AF) is a powerful risk factor for stroke, independently increasing risk fivefold in all age ranges. Radio frequency (RF) energy, a procedure that is used in many clinical methods and studies [1,2,3] to treat arrhythmia, is applied around the pulmonary veins (PVs) using a catheter to block sources of ectopic foci. This minimally invasive arrhythmia procedure is performed by matching the anatomical landmarks manually annotated in the pre-scanned three-dimensional (3D) computed tomography angiography (CTA) image with the rough points obtained in the real-time electrophysiology (EP) catheterization and form the atrial shape [4]. However, since it is time-consuming to designate the 3D landmark manually, an automatic method for localization of the target objects is needed to reduce the time required and improve the accuracy of the procedure.

The left atrial appendage (LAA) is known as one of the major locations of cardiac thrombus formation. LAA occlusion devices are used to prevent stroke in AF patients [5], and accurate measurement of the LAA diameter is important for determining the appropriate device sizes. However, as LAA can have a variety of sizes and shapes, it is challenging to describe its anatomic structure. Localization of the LAA is a prerequisite for automatically finding a longitudinal view of the LAA, where the operator can define the size of the device. Furthermore, in fluid simulations based on the left atrium for predicting patient-specific blood patterns or planning LAA intervention, precise manual annotation of in- and out-lets such as four PVs and LAA is essential [6].

A benchmark for a number of left atrium (LA) segmentation algorithms was reported [7,8]. Because of variations in the intensities and shapes of the PVs, the LAA, and other anatomical structures surrounding the LA, many different approaches have been proposed for segmenting 3D medical images. Purely data-driven methods using region growing and graph cut as well as more advanced methods using prior knowledge were proposed in [9,10]. Region growing based approaches need a manual placement of the seed point inside the LA. There are atlas-based approaches, which use deformable registration to propagate an atlas to the images [11,12,13]. In addition, multi-atlas based approaches have been introduced [14,15,16]. Qiao et al. proposed a multi-atlas registration method based on the contrast probability map [15]. Nuñes-Garcia et al. utilized rank similarity of different atrial shapes for multi-atlas segmentation [16]. Recently, deep convolutional neural network (CNN)-based methods for LA segmentation have been proposed [17,18,19,20]. CNN automatically extracts features and outperforms traditional methods. They show higher accuracies on LA segmentation tasks and may be used for quantification or the prerequisite process of LA landmark detection. Recently, other approaches were introduced using CNN to find landmarks [21,22]. An initialized position is updated by regression and classification using small patches [21]. In addition, multi-scale deep reinforcement learning successfully finds anatomical landmarks [23], which reduces computational complexity by policy-based searches. However, sometimes the multi-atlas or the labeled images are often difficult to locate or generate, especially in clinical image datasets.

In morphometrics, the anatomical landmark positions can be analyzed using various statistical techniques independently of size, position, and orientation so that the only variables being observed are based on morphology. Various applications based on geometric morphometrics are also introduced [24,25,26,27]. A fully automatic method is introduced [28] for simultaneously localizing two target clinical regions in CTA images using pair-wise estimation based on their geometric relationships. Similarly, coronary arteries are also identified successfully using the geometric estimation [29].

**Figure 1:**
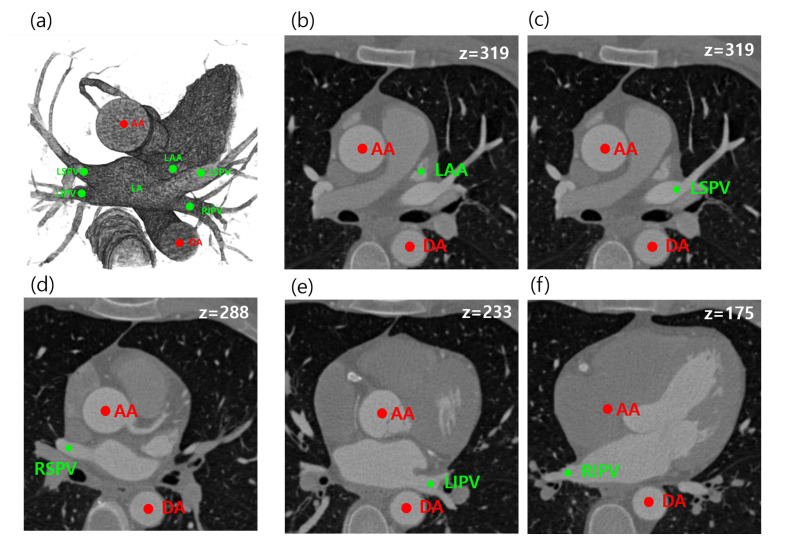
Relationships of five target (**green**) and two reference (**red**) objects in computed tomography angiography (CTA) images are illustrated. All the left atrial landmarks are visualized in 3D volume rendering (**a**), and each target object and the reference objects (AA and DA) are separately visualized at a specific 2D slice of *z* (**b**–**f**). LSPV, left superior pulmonary vein; LIPV, left inferior pulmonary vein; RSPV, right superior pulmonary vein; RIPV, right inferior pulmonary vein; LAA, left atrial appendage; CTA, computed tomography angiography.

In this paper, we propose a fully automated method for the localization of multiple target regions using relative geometric priors from reference objects, which improves the geometric method. The proposed method can automatically localize a total of five anatomical objects in cardiac CT, which are needed for arrhythmia procedures as well as for assisting an image-guiding LAA occlusion procedure. The main targets are left superior pulmonary vein (LSPV), left inferior pulmonary vein (LIPV), right superior pulmonary vein (RSPV), right inferior pulmonary vein (RIPV), and LAA. As reference objects, the ascending aorta (AA) and descending aorta (DA) are considered for geometric estimation. The target and reference objects in CTA images are shown in Figure 1. In summary, we provide three main contributions:

- We proposed an adaptive model for estimating patient-specific image parameters.
- We designed a Bayesian formulation utilizing the relative geometric prior distribution to solve the LA landmark detection problem.
- We estimated and provided the relative prior distributions between five anatomies (LSPV, RSPV, LIPV, LSPV, LAA) and AA and DA based on distance measures.

We organize the rest of this paper as follows. We describe a Bayesian formulation that comprises the entire process of our method in Section 2, and the details are described in Section 2.1, Section 2.2 and Section 2.3. Experimental results are presented in Section 3. Finally, the discussion and conclusion are drawn in Section 4.

## 2. Materials and Methods

**Figure 2:**
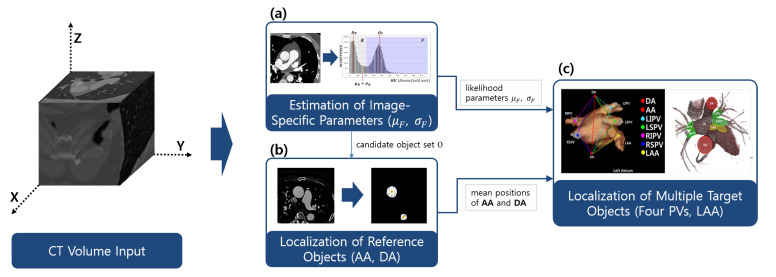
Workflow of the proposed method: image-specific parameters are estimated for the adaptation of various CTA images with different intensity distributions (**a**). Then, the ascending aorta (AA) and descending aorta (DA) are used to localize other anatomies, including four PVs, and LAA (**b**,**c**). PVs, pulmonary artery; LAA, left atrial appendage.

The proposed method simultaneously estimates multiple objects on the basis of anatomic and geometric features. Our target objects are four PVs and LAA. As these have variable shapes and locations, it is difficult to automatically localize the target objects if each object is considered separately. However, by considering geometric information from the aorta as a robust reference, robust detection of the PVs and LAA is possible. The overall workflow was divided into three parts, as shown in Figure 2. CTA images were observed to have a large variation in intensity distribution. Hence, adaptive image parameters needed to be estimated before the next processes Figure 2a. The image parameters were utilized for the two localization processes both for reference and target objects Figure 2b,c.

We can obtain the geometric prior distributions of the five targets by estimating the AA and DA together to derive the geometric information in terms of angle and distance. We can estimate AA, DA, PVs, and LAA automatically by using the corresponding geometric information as the prior distribution.

After pre-processing, we assume the set of objects $O=\{C_{i}\;|\;1\leq i\leq N,N\geq m+n\}$, where *m* is the number of target objects, and *n* is the number of reference objects. Note that $C_{i}$ is either a voxel or a discrete region depending on the pre-processing method. Our goal is to find m+n objects among *N* candidates. Here we assume that *m* target objects are conditionally independent of the reference objects. Then, for a given image *I*, we have the following Bayesian formula:

$$\begin{array}{cc} & P(\underset{\mathrm{target}\;\mathrm{objects}}{\underset{︸}{C_{1},\ldots ,C_{m}}},\underset{\mathrm{reference}\;\mathrm{objects}}{\underset{︸}{C_{m+1},\ldots ,C_{m+n}}}|I)\\ & \propto P\left(I|C_{1},\ldots ,C_{m}\right)\cdot P\left(I|C_{m+1},\ldots ,C_{m+n}\right)\;\cdot P\left(C_{1},\ldots ,C_{m},C_{m+1},\ldots ,C_{m+n}\right), \end{array}$$

where P(I) is treated as a constant since *I* is given data. If we set the probability of the reference objects as $P_{mn}:=P\left(C_{m+1},\ldots ,C_{m+n}\right)$, then we have

$$\begin{array}{c} P\left(C_{1},\ldots ,C_{m},C_{m+1},\ldots ,C_{m+n}\right)=P\left(C_{1},\ldots ,C_{m}|C_{m+1},\ldots ,C_{m+n}\right)\cdot P_{mn}. \end{array}$$

Putting Equation (1) into Equation (1), we now have

(1) $$\begin{array}{cc} & P(C_{1},\ldots ,C_{m},C_{m+1},\ldots ,C_{m+n}|I)\\ & \propto P\left(I|C_{1},\ldots ,C_{m}\right)\cdot P\left(I|C_{m+1},\ldots ,C_{m+n}\right)\cdot P\left(C_{1},\ldots ,C_{m}|C_{m+1},\ldots ,C_{m+n}\right)\cdot P_{mn}. \end{array}$$

The probabilities of both target and reference objects are simultaneously modeled by Equation (3). However, we separately consider target- and reference-associated terms for reducing computational complexity. Thus, we first identify the reference objects by

(2) $$\begin{array}{c} \begin{array}{cc} & \underset{C_{m+1},\ldots ,C_{m+n}}{arg max}P\left(I|C_{m+1},\ldots ,C_{m+n}\right)\cdot P_{mn}\\ & =\underset{C_{m+1},\ldots ,C_{m+n}}{arg max}L\left(I|C_{m+1},\ldots ,C_{m+n}\right)+L\left(C_{m+1},\ldots ,C_{m+n}\right), \end{array} \end{array}$$

where L(·) is the log-likelihood, logP(·). After the reference objects $C_{m+1}^{*},\ldots ,C_{m+n}^{*}$ are detected, the probability associated with the reference objects, $P\left(I|C_{m+1}^{*},\ldots ,C_{m+n}^{*}\right)\cdot P_{mn}^{*}$ is assumed to be a uniform distribution. Thus, the multiple target objects are finally estimated by the equation

(3) $$\begin{array}{c} \begin{array}{c} \underset{C_{1},\ldots ,C_{m}}{arg max}P\left(I|C_{1},\ldots ,C_{m}\right)\cdot P\left(C_{1},\ldots ,C_{m}|C_{m+1}^{*},\ldots ,C_{m+n}^{*}\right)\\ =\underset{C_{1},\ldots ,C_{m}}{arg max}L\left(I|C_{1},\ldots ,C_{m}\right)+L\left(C_{1},\ldots ,C_{m}|C_{m+1}^{*},\ldots ,C_{m+n}^{*}\right). \end{array} \end{array}$$

In Section 2.2 and Section 2.3, we analyze each component in detail.

### 2.1. Estimation of Image-Specific Parameters

**Figure 3:**
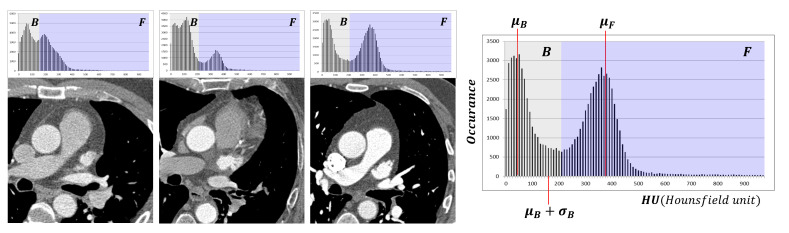
Various intensity histograms showing how the image can be parsed. The mean intensity of contrast regions ($\mu_{F}$) and the mean intensity of non-contrast regions ($\mu_{B}$) are adaptively estimated using k-means clustering. Foreground (F) and background (B) indicate the contrast regions and non-contrast regions, respectively. The display window level and width for the images in this figure are fixed to 60 and 700 HU, respectively, for visualization.

The PVs, LAAs, AA, and DA all have the common feature that they belong to contrast-enhanced regions. However, since contrast density may vary from case to case, as shown in Figure 3, we propose a method to adaptively determine thresholds by automatically separating foreground and background values using the unsupervised manner from the input images.

First, we analyze the histogram of input images after thresholding by 0 HU. Then, $R^{+}$ is divided into the foreground set (F ) and the background set (B) by applying k-means clustering (k=2) with the within-cluster sum-of-squares (WCSS) optimization function.

Let $\mu_{B}$ and $\sigma_{B}$ be the average and standard deviation, respectively, of the set {I(x,y)∣I(x,y)∈B}. Likewise, let $\mu_{F}$ and $\sigma_{F}$ be the average and standard deviation, respectively, of the set {I(x,y)∣I(x,y)∈F}. Then, the input image is thresholded to generate candidates for the reference and targets, and the resulting mask is generated as follows:

(4) $$O:=[I_{1}\leq I\left(x,y\right)\leq I_{2}],$$

where $I_{1}=\min\left(\mu_{B}+\sigma_{B},\mu_{F}-\sigma_{F}\right)$ and $I_{2}=\mu_{F}+\sigma_{F}$. The estimated values will also be used for the target value of the likelihood function in Section 2.3.

### 2.2. Localization of Reference Objects

The ascending and descending aorta (AA and DA) have the relatively distinct features of large, thick, stem-like shapes and the appearance of two large circles in the axial plane. For this reason, AA and DA are selected as reference objects (n=2). The locations of AA and DA can be robustly identified by the method in [28], which exploits the ratios of the eigenvalues, as their shape is relatively close to circular in the axial view. To find the eigenvalues, first, the resulting mask in Equation (4) is labeled as multiple discrete regions using connected-component analysis (CCA):

(5) $$\begin{array}{c} O=\{C_{i}|1\leq i\leq N\},\;\mathrm{where}\;N\geq 2. \end{array}$$

Then, we consider the covariance matrix $\Sigma_{C_{i}}$ for each $C_{i}$:

(6) $$\begin{array}{c} \begin{array}{c} \Sigma_{C_{i}}=\left[\begin{array}{cc} \sigma_{xx,C_{i}}^{2} & \sigma_{xy,C_{i}}^{2}\\ \sigma_{yx,C_{i}}^{2} & \sigma_{yy,C_{i}}^{2} \end{array}\right] \end{array} \end{array}$$

where $\left(x_{k},y_{k}\right)\in C_{i}$. Let $\lambda_{1,i}\leq \lambda_{2,i}$ be the eigenvalues of $\Sigma_{C_{i}}$ and let $r_{i}:=\lambda_{1,i}/\lambda_{2,i}$. Then, the ideal value of $r_{i}\approx 1$ if the object $C_{i}$ is near circular. The joint log-likelihood for the two most isotropic components is formulated as

(7) $$\begin{array}{c} \left.\begin{array}{cc} L(I|C_{m+1},C_{m+2}) & =-\frac{|1-r_{m+1}{|}^{2}+{\left|1-r_{m+2}\right|}^{2}}{\sigma_{r}^{2}} \end{array}\right. \end{array}$$

where $\sigma_{r}^{2}$ is the variance of the eigenvalue ratio for AA and DA. The log-likelihood measures the deviation of the two components from the isotropy for both AA and DA.

**Figure 4:**
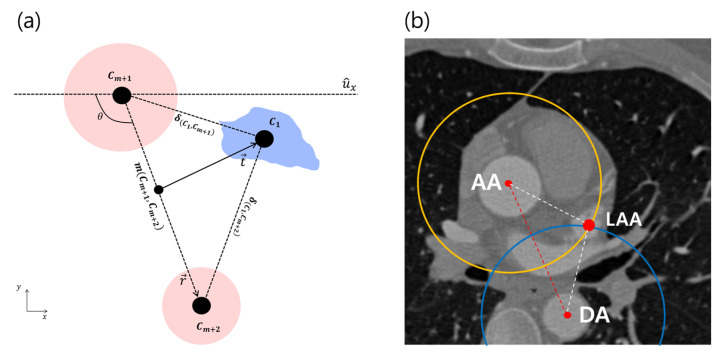
A geometric model for AA and DA ($C_{m+1},C_{m+2}$) and a target ($C_{1}$). (**a**) The distance $|\vec{r}|$ and angle between $\vec{r}$ and ${\hat{u}}_{x}$ are sampled to obtain a prior distribution for reference, where $\vec{r}$ is the vector between the two references. *m* is the mean point between the two references, and ${\vec{t}}_{1}=C_{1}-m$ is the direction of the target $C_{1}$, which would be used to specify a single solution. δ is the distance between $C_{1}$ and $C_{m+k}$. (**b**) The simplified geometry is overlaid on a CTA image with an example of $C_{1}$ = LAA. More details about the intersection of two circles whose centroids are AA and DA are described in Section 3.3. AA, ascending aorta; DA, descending aorta; LAA, left atrial appendage.

The prior term with two references is denoted as $L(C_{m+1},C_{m+2})$. With the discrete candidates in Equation (5), the geometric features of the aorta in the axial images are described in terms of the distance δ and the angle θ between the ascending and descending aorta (Figure 4):

(8) $$\begin{array}{c} L\left(C_{m+1},C_{m+2}\right)=L\left(\theta_{C_{m+1},C_{m+2}}\right)+L\left(\delta_{C_{m+1},C_{m+2}}\right). \end{array}$$

This prior term $L(C_{m+1},C_{m+2})$ is interpreted as the two learned prior distributions with regard to the angles and distances. Let ${\vec{r}}_{C_{m+1},C_{m+2}}$ be the vector from the centroid of $C_{m+1}$ to the centroid of $C_{m+2}$, and let $\delta_{C_{m+1},C_{m+2}}$ be its norm. The variable $\theta_{C_{m+1},C_{m+2}}$ is the angle between the *x*-axis and ${\vec{r}}_{C_{m+1},C_{m+2}}$. Let $\bar{\theta }$ and $\bar{\delta }$ be the mean angle and distance, where the distance and angle variations can be evaluated as the ratios $\frac{\delta_{C_{m+1},C_{m+2}}}{\bar{\delta }}$ and $\frac{\theta_{C_{m+1},C_{m+2}}}{\bar{\theta }}$, respectively. The two independent variables are converted into the prior probabilities as

(9) $$\begin{array}{c} L\left(C_{m+1},C_{m+2}\right)=-\left(\frac{|1-\delta_{C_{m+1},C_{m+2}}/\bar{\delta }{|}^{2}}{\sigma_{\delta }^{2}}+\frac{|1-\theta_{C_{m+1},C_{m+2}}/\bar{\theta }{|}^{2}}{\sigma_{\theta }^{2}}\right). \end{array}$$

The ratios $\frac{\delta_{C_{m+1},C_{m+2}}}{\bar{\delta }}$ and $\frac{\theta_{C_{m+1},C_{m+2}}}{\bar{\theta }}$ are a perfect match to our model. Recall the reference estimator in Equation (2); two reference objects are found by maximizing the following equation:

(10) $$\begin{array}{c} \begin{array}{c} \underset{C_{m+1},C_{m+2}}{arg max}L\left(I|C_{m+1},C_{m+2}\right)+L\left(C_{m+1},C_{m+2}\right). \end{array} \end{array}$$

**Figure 5:**
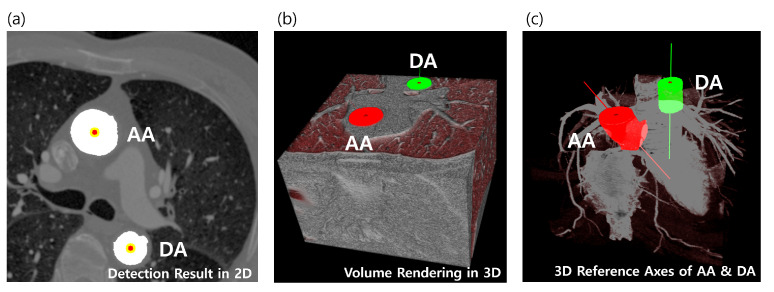
The detected references are visualized both in 2D and 3D (**a**,**b**). Each cylinder-like object is approximated to a vector to cover the entire range of volume (**c**). AA, ascending aorta; DA, descending aorta.

The object pair $C_{m+1}^{*}$ and $C_{m+2}^{*}$, which maximize Equation (10), is assumed to be AA and DA. We now have two robust cylinder-like objects that cover the entire range of the CTA volume as patient-specific references, as shown in Figure 5.

### 2.3. Localization of Multiple Target Objects

Although the deviation of the geometric relationships between the clinical positions from the average is small, the variation in the size of the whole heart can be very large, for example between adults and children. In addition, the angle at which the heart is placed may be slightly different in different cases. To minimize this variance, we used a prior distribution created by sampling the distance ratios between each clinically named location and the references, similarly to the flexible prior distribution illustrated. This can fit most human heart shapes if the reference positions are correctly posed (such as by using the robust reference identification method introduced in Section 2.2).

Unlike the reference objects, the boundaries of the target objects are generally not clear. Hence, it may not be possible to have discrete components that were used in the technique for identifying the reference objects. Instead, we propose a new feature for each pixel using the relative distances.

Let $\left(x_{m+1},y_{m+1}\right)$ and $\left(x_{m+2},y_{m+2}\right)$ be the coordinates of the centroids of $C_{m+1}$ and $C_{m+2}$, respectively. We define three distances by

(11) $$\begin{array}{ccc} d_{m+1}\left(x,y\right) & = & \sqrt{{\left(x-x_{m+1}\right)}^{2}+{\left(y-y_{m+1}\right)}^{2}},\\ d_{m+2}\left(x,y\right) & = & \sqrt{{\left(x-x_{m+2}\right)}^{2}+{\left(y-y_{m+2}\right)}^{2}},\\ d_{m+1,m+2} & = & \sqrt{{\left(x_{m+1}-x_{m+2}\right)}^{2}+{\left(y_{m+1}-y_{m+2}\right)}^{2}}. \end{array}$$

As we have the prior distributions of the relative distances between all target objects, we denote $\mu_{i,j}$ and $\sigma_{i,j}^{2}$ to be the average distance between the *i*-th object and the *j*-th object, and its variance, respectively.

Then, we propose a target similarity measure for each target $C_{k}$ as follows:

(12) $$\begin{array}{cc} & f_{k}\left(x,y\right)=L\left(d_{m+1,k}\right)+L\left(d_{m+2,k}\right)\\ & :=-\frac{|d_{m+1,m+2}/\mu_{m+1,m+2}-d_{m+1}\left(x,y\right)/\mu_{k,m+1}{|}^{2}}{\sigma_{k,m+1}^{2}}-\frac{|d_{m+1,m+2}/\mu_{m+1,m+2}-d_{m+2}\left(x,y\right)/\mu_{k,m+2}{|}^{2}}{\sigma_{k,m+2}^{2}}. \end{array}$$

The target similarity measure is computed for every 2D slice. Our estimated parameter for reference $\mu_{m+1,m+2}$ is 81.6 mm. Table 1 shows our estimated parameter values from the distance measurement samples by Equation (11), which can be referred for Equation (12).

**Figure 6:**
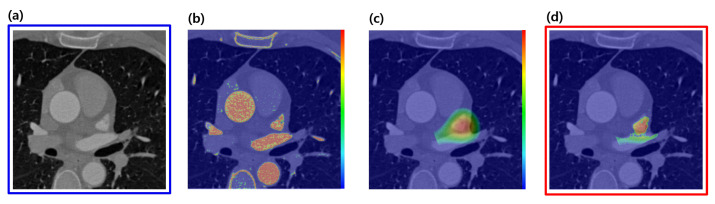
Example of a process for specifying a target object LAA in axial images; (**a**) original image; (**b**) likelihood, $L(I|C_{1},\ldots ,C_{m})$; (**c**) prior $f_{k}\left(x,y\right)$; (**d**) posterior, $\max_{1\leq k\leq m}f_{k}\left(x,y\right)$; LAA, left atrial appendage.

**Figure 7:**
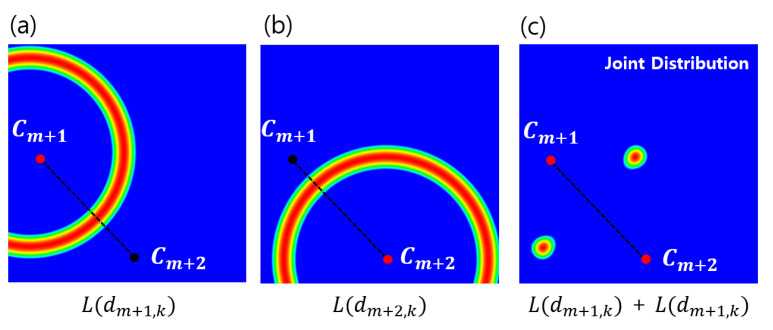
Visualization of geometric prior distributions: the distance-based PDFs based on two reference locations, $C_{m+1}$ (**a**) and $C_{m+2}$ (**b**). While each ring-shaped PDF is not able to identify a unique location, the joint PDF (**c**) can simply designate a target location; PDF, probability density function.

**Figure 8:**
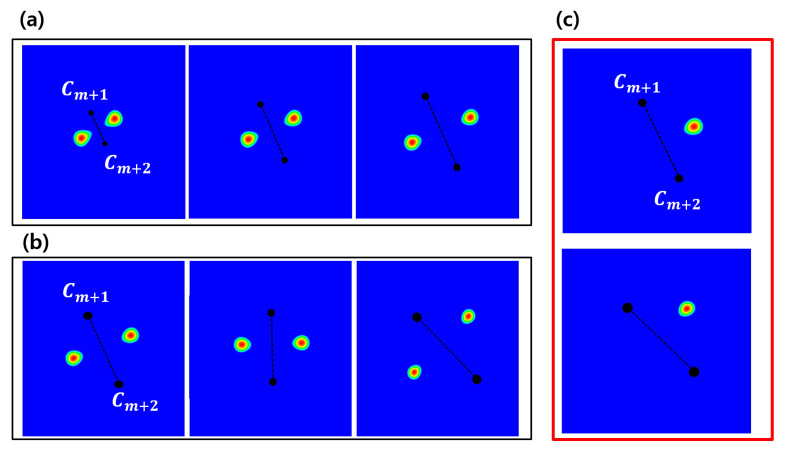
Flexibility of prior distributions are shown in terms of (**a**) scale and (**b**) rotation by varying distance δ and angle θ of reference $\vec{r}$. (**c**) Prior distribution defined by two distance samples may give two peaks, one for the correct target and the other on the opposite side. The distribution on the unwanted opposing side is removed simply by using the direction $\vec{t}$.

Given a robust reference, a relative distance distribution is determined in order to localize the target object, as shown in Figure 6c. Additionally, the elements of $f_{k}\left(x,y\right)$ are separately analyzed, and Figure 7 shows why distance distributions from both references are needed to localize an object. However, since the intersection of each distribution forming the 2D ring shape appears in two places, it still does not uniquely specify a single position. Therefore, we combine it with the directional information $\vec{t}$ shown in Figure 4a to obtain the final distribution. The behavior of $f_{k}\left(x,y\right)$ in Equation (12) is also analyzed to show that the target object can be localized without depending on the rotation and scale by using the geometric prior distribution, which changes dynamically according to changes in the position of the reference, as shown in Figure 8.

**Figure 9:**
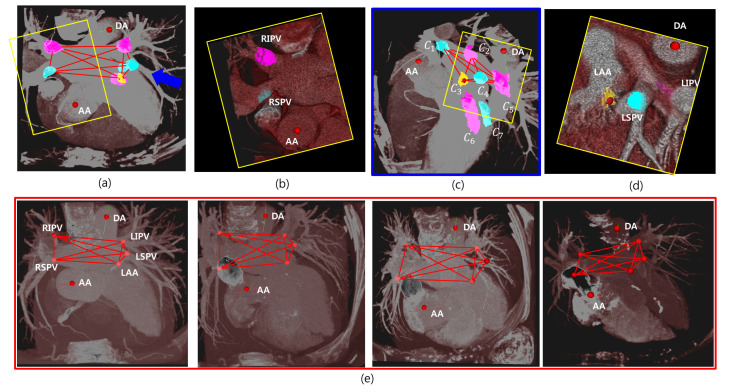
Results and visualizations of the candidates and their estimation. (**a**) Five anatomies are visualized. (**b**) The magnified view of RIPV and RSPV. (**c**) The rotated view of the results. Some candidate objects are co-visualized. Simple level comparison enables one to select the correct objects. (**d**) The magnified views of LSPV, LIPV, and LAA. (**e**) The results of four example cases. LSPV, left superior pulmonary vein; LIPV, left inferior pulmonary vein; RSPV, right superior pulmonary vein; RIPV, right inferior pulmonary vein; LAA, left atrial appendage.

In order to simultaneously localize multiple targets in 3D, we compute $f_{k}$ for each 2D slice, and we finally obtain the following 3D mask by thresholding the resultant measure:

(13) $$\begin{array}{c} \Omega =\left[\Omega_{k}|\max_{1\leq k\leq m}f_{k}\left(x,y\right)\geq \alpha \right], \end{array}$$

where the element $\Omega_{i}$ is a grouped sample set specified by Equation (13), which is visualized, for example, in Figure 9c.

A common fact that the target objects exist in the contrast region is considered in the likelihood function for the targets, which is formulated as

(14) $$\begin{array}{c} L\left(I|C_{1},\ldots ,C_{m}\right)\approx L\left(I|\Omega_{1},\ldots ,\Omega_{m}\right)=\sum_{k=1}^{m}-\frac{|\mu_{F}-I_{k}{|}^{2}}{\sigma_{F}^{2}}, \end{array}$$

where $I_{k}$ is the mean intensity of all voxels in $C_{k}$. The image-specific parameters, $\mu_{F}$ and $\sigma_{F}$, for Equation (14), can be calculated by the adaptive method given in Section 2.1. The higher probabilities are mapped on the contrast regions by computing the likelihood as shown in Figure 6b.

Then, the positions of the target objects with unclear boundaries can be specified by the posterior probability in Figure 6d.

Subsequently, we formulate the probability of the target objects as follows:

(15) $$\begin{array}{c} L\left(\Omega_{1},\ldots ,\Omega_{m}|C_{m+1},\ldots ,C_{m+n}\right)=\sum_{1\leq i<j\leq m}-\frac{|\mu_{i,j}-\delta_{i,j}{|}^{2}}{\sigma_{i,j}^{2}}, \end{array}$$

where $\delta_{i,j}$ is the distance between $\Omega_{i}$ and $\Omega_{j}$, and $\mu_{i,j}$ and $\sigma_{i,j}^{2}$ are its average and variance, respectively, for Equation (15). The geometry of the relationships among the components is similar to a complete graph. The goal of our method is to find a geometry that fits our prior probabilities.

## 3. Experimental Results

We have performed a number of experiments to demonstrate the robustness of our system and the quantitative results for the five target objects.

### 3.1. Data Set

The proposed method was applied on a total of 60 CTA images. Besides thirty-two public datasets, Rotterdam Challenge images [30,31], which are well focused on hearts, were acquired on the CT scanners from various vendors. We additionally selected twenty-eight cases with variations in the field of views, contrast injection timings, artifacts, and morphology from Severance Hospital, Republic of Korea. Each CTA image was reconstructed to 512 × 512 × [272, 433] voxels with an isotropic voxel size between (0.26 mm × 0.26 mm × 0.26 mm) and (0.48 mm × 0.48 mm × 0.48 mm ). Ground truth sets for 60 CTA cases were manually labeled by a medical expert. We have each labeled target region (four PVs and LAA).

### 3.2. Parameters

We fixed parameters for Equation (9) as $\bar{\delta }=81.6$ mm, $\bar{\theta }=66.9^{\circ }$, $\sigma_{\delta }=0.132$, and $\sigma_{\theta }=0.011$. The parameters were referred from the previous investigation on aortic localization [28], which were measured from CTA images from eight patients in the Rotterdam Challenge training set [30,31]. $\sigma_{\delta }$ and $\sigma_{\theta }$ are the parameters normalized by $\bar{\delta }$ and $\bar{\theta }$, which play the roles of the internal weighting parameters that can be adjusted so that the larger the difference from the mean values ($\bar{\delta }$, $\bar{\theta }$), the greater the penalty. Despite the limited amount of data, the estimated average values can be converged quite closely to the actual values. For example, the average arch width (notated as $\bar{\delta }$) has been reported as 82 mm, which is a value obtained from 234 patient cases in a clinical study [32].

The parameters of Equation (12) were set to the values of a geometric Gaussian distribution in Table 1. The parameters are newly investigated in this paper with the same dataset from eight patient’s CTA images, using the Rotterdam Challenge training dataset [30,31].

### 3.3. Analysis of Geometric Prior Distribution

Given the robust reference objects, a geometric prior distribution was determined to localize a target object. Figure 7 shows why two reference objects are needed to localize a target position. The elements of $f_{k}\left(x,y\right)$ are separately analyzed, and each 2D ring-shaped distribution is drawn in Figure 7a,b based on $C_{m+1}$ and $C_{m+2}$. For the experiment, the learned parameters, which are provided in Table 1, are referred for localizing the five target objects based on AA and DA.

Figure 8 shows that the geometric prior distributions were rotation- and scale-invariant. A target object can be localized regardless of rotation and scale using the geometric prior distribution that flexibly changes according to the change of the positions of the reference objects, as shown in Figure 8a,b. However, since the joint distributions $L\left(d_{m+1,k}\right)+L\left(d_{m+2,k}\right)$ have two local peaks, it still can not specify a unique target position. Hence, we used directional information $\vec{t}$ described in Figure 8c together for a unique position.

### 3.4. Quantitative Comparison with Other Methods

Table 2 presents the results of localization of the five targets, a quantitative comparison of the proposed method with the result given by a workstation (Vitrea, by Vital Images [33]). We used the following measures to evaluate the robustness:

- TPR, true positive rate ($=\frac{\mathrm{TP}}{\mathrm{TP}+\mathrm{FN}}$);
- TP, true positive; TP:=|x∈Y|;
- FN, false negative; FN:=|Y|−|x∈Y|;

where *X* and *Y* can be the outputs by one of methods and ground truth, respectively. In terms of a detection rate, TPR is computed to compare the robustness, as shown in Table 2. Even though the experiments were done without any user interaction or parameter changes, the reference objects, AA and DA, were detected 100% of the time in our dataset.

Table 2 presents the results for PVs and LAA localizations. The quantitative comparison of our method with the output given by the CT EP planning function of a workstation (Vitrea, by Vital Images [33]) is shown. The success rate was measured with a prepared ground truth set corresponding to 60 images in the dataset we tested.

Our proposed method required 7.34 s total computation time using an i7 3.50-GHz 32-GB PC. Most of the time was consumed in the processing the object preparation. Vitrea required about 10–20 s computation time, depending on CTA images, using dual Xeon E5-2690 2.90 GHz 128-GB HP workstation. Vitrea does not provide a description of the detailed methodology for the detection of LA and its landmarks, but it seems to always conduct LA segmentation first, and then the landmark detection process is done based on the LA segment. On the other hand, the proposed method did not need a segmentation process for LA, rather it could directly detect LA landmarks, which seems to make a difference in computation time.

Figure 9 shows some examples, some target candidates, and their estimated geometries, where maximum probability is visualized with maximum intensity projection (MIP), in the axial view in Figure 9a and a magnified view in the yellow box in Figure 9b. It shows the candidates on the target regions, RIPV and RSPV. All the candidates Ω in Equation (13) are visualized in Figure 9c from a view slightly rotated to the blue arrow in Figure 9a. Figure 9d shows a magnified view of the yellow box in Figure 9c. In this example, the five objects connected to each other with red edges are the estimated 3D target objects among seven candidate objects by Equation (15), and $C_{1},C_{2},C_{3},C_{4}$, and $C_{5}$ are RSPV, RIPV, LAA, LSPV, and LIPV, respectively. Figure 9e demonstrates that the results were detected with maximum probabilities from several cases. We find such geometries form a complete graph, and its vertices are our target objects.

## 4. Discussion and Conclusions

In this paper, we proposed a Bayesian framework to localize five target objects (four PVs and LAA) in LA based on the two reference objects (AA and DA) by designing a Bayesian formulation that utilizes geometric prior distributions. The proposed method is based on the important prior knowledge that there are geometrical relationships among the cardiac anatomies and that follow Gaussian distributions. Multiple target objects whose structures are complex can be robustly localized. For successful localization of the multiple objects, first, we propose an adaptive model for estimating patient-specific image parameters using an unsupervised manner, which can be generally utilized for the preprocessing of other methods. We also designed a Bayesian formulation comprising the relative geometric prior distribution to solve the LA landmark detection problem. We investigated and provided the geometric prior distributions between five anatomies (LSPV, RSPV, LIPV, LSPV, and LAA) and AA and DA based on distance measures. As a result, the average detection rates were 1.0, 0.97, 0.91, 1.0, and 0.94 for localizing LSPV, LIPV, LAA, RIPV, and RSPV, respectively. The proposed method required a total of 7.34 s computation time.

The geometric prior distributions can be easily integrated into other methods. In addition, the proposed method can be easily applied to other applications since it can specify all positions in the cardiac CT image based on two robust reference objects. For example, policy-based localization methods [23,34,35] using deep reinforcement learning, which are the new approaches in the literature, are very fast compared to other approaches that require an extensive search. However, in order to track a target object from a random location in CT images, the direction toward the target location is sequentially searched using only local boxed information. For this reason, sometimes it fails to converge to the target location. Such a problem may occur because the agent cannot use the context of the global heart structure. Initializing the start position of the agent using the proposed method can help to minimize the failure case.

A limitation of the proposed method may arise when the reference or target anatomic parts are abnormally positioned. Cardiac malposition is very rarely reported, but the position of the heart can be reversed, or parts may be missing as in cases of situs inversus, asplenia, or polysplenia. However, we will include case-specific distributions in our database, which is supposed to solve these problems.

In future work, we plan to approximate the likelihood function using a convolutional neural network to obtain a more robust posterior probability in order to improve the robustness of the proposed method, and the geometric prior distribution will be combined with a policy-based method to minimize the failure rate of target object localization in further research. In addition, we aim to apply the proposed method to automatically analyze the morphology of LAA, select device sizes for the LAA procedure, and automate the detection of the landing zone. For LA fluid simulations, we also plan to generate simulation-ready LA geometries by automatically localizing the inlets and outlets of blood flow.

## Figures and Tables

**Table 1:** Estimated distances between reference and target objects as optimal internal parameters for localization of four pulmonary veins and the left atrial appendage.

Reference	LSPV (mm)	LIPV (mm)	RSPV (mm)	RIPV (mm)	LAA (mm)
	μ σ	μ σ	μ σ	μ σ	μ σ
**AA**	65.71 2.95	80.09 6.04	44.16 3.55	63.74 8.71	49.23 3.02
**DA**	46.54 7.31	26.22 4.38	82.15 9.62	63.31 8.66	65.37 10.77

μ: average distance, σ: deviation where $\mu =\frac{1}{n}\sum_{i=1}^{n}\delta_{i}$, $\sigma =\sqrt{\frac{\sum_{i=1}^{n}{\left(\mu -\delta_{i}\right)}^{2}}{n-1}}$. LSPV, left superior pulmonary vein; LIPV, left inferior pulmonary vein; RSPV, right superior pulmonary vein; RIPV, right inferior pulmonary vein; LAA, left atrial appendage.

**Table 2:** Quantitative comparison for the detection rate of the five target locations from 60 CTA images, where TPR is the measurement.

Method	LSPV	LIPV	LAA	RIPV	RSPV	Average
Proposed (Public [31])	1.0	1.0	0.93	1.0	0.96	0.98
Proposed (Selected)	1.0	0.94	0.88	1.0	0.92	0.95
Vitrea (Public [31])	0.94	0.97	-	0.97	0.81	0.92
Vitrea (Selected)	0.93	0.89	-	0.96	0.82	0.90

Note that LAA could not be directly compared since Vitrea does not provide an automatic LAA detection function. TPR, true positive rate $=\frac{\mathrm{True positive}}{\mathrm{True Positive}\;+\;\mathrm{False Negative}}$; LSPV, left superior pulmonary vein; LIPV, left inferior pulmonary vein; RSPV, right superior pulmonary vein; RIPV, right inferior pulmonary vein; LAA, left atrial appendage.

## Data Availability

Not applicable.
